# Calcipotriol/Betamethasone Dipropionate Aerosol Foam for Plaque Psoriasis: A Prospective, Observational, Non-Interventional, Single-Center Study of Patient Adherence and Satisfaction in Daily Use

**DOI:** 10.5826/dpc.1103a56

**Published:** 2021-05-20

**Authors:** Francisco José Navarro-Triviño, Mario Lozano-Lozano, Ricardo Ruiz-Villaverde

**Affiliations:** 1 Department of Contact Eczema and Immunoallergic Diseases, Hospital Universitario San Cecilio, Granada, Spain; 2 Department of Physical Therapy, Faculty of Health Sciences, and University of Granada & Sport and Health Joint University Institute (iMUDS), Granada, Spain; 3 Department of Psoriasis, Dermatology, Hospital Universitario San Cecilio, Granada, Spain

**Keywords:** adherence, satisfaction, calcipotriol, betamethasone, aerosol foam, psoriasis

## Abstract

**Background:**

Psoriasis is a chronic inflammatory disease that has a negative impact on patients’ quality of life. Patients with mild–moderate psoriasis can be treated with topical medications, such as the combination drug calcipotriol/betamethasone dipropionate (Cal/BD).

**Objectives:**

This study investigated the adherence of psoriasis patients to therapy with Cal/BD aerosol foam, as well as their satisfaction with the treatment’s efficacy, safety, and effect on their quality of life.

**Methods:**

Patients with mild–moderate plaque psoriasis were eligible to participate in this open-label, non-placebo controlled, prospective single-center study. Adherence to treatment was assessed using the Morisky-Green scale 4 and 12 weeks after the start of treatment. Satisfaction with the treatment was assessed using the abbreviated Treatment Satisfaction Questionnaire for Medication (TSQM-9). The severity of psoriasis was assessed on the mIGA and PGA scales, and the impact on quality of life was assessed using the PDI and DLQI scales.

**Results:**

A total of 65 patients entered the study. Adherence to treatment was good, with 73.8% of patients showing high adherence at 12 weeks. Satisfaction was also good, with 46 patients (70.8%) being completely satisfied.

**Conclusions:**

Over a 4-week period, patients treated with Cal/BD aerosol foam had significant improvement in disease severity that was directly related to treatment adherence.

## Introduction

Psoriasis is a chronic inflammatory disease that affects 2%–4% of the western population [1]. It is characterized by the formation of well-defined erythematous-squamous plaques and a complex immune mechanism [2]. Psoriasis is recognized by the World Health Organization as a debilitating, painful disease [3]. The association of comorbidities such as psoriatic arthropathy and the risk of cardiovascular disease make psoriasis a systemic disease. The disease has a psychological impact on patients, who have a greater risk of depression, anxiety, and even social stigmatization [4]. All of these have a significant negative impact on the patients’ quality of life.

The development in recent years of biological drugs has revolutionized the treatment of psoriasis. However, topical medications are considered the cornerstone of therapy for many patients [5]. Patients with a score less than 10 on a psoriasis severity index (eg, PASI, BSA, or DLQI) can be treated with topical monotherapy. Patients with moderate plaque psoriasis who were treated with calcipotriol/betamethasone dipropionate (Cal/BD) aerosol foam for 4 weeks achieved a Psoriasis Area and Severity Index (PASI) score of 75, which was higher than that achieved with 12-week methotrexate and acitretin therapy but similar to that with fumaric acid esters [6]. This finding reaffirms the indication for topical treatment in patients with mild–moderate plaque psoriasis.

Current guidelines recommend the topical use of a vitamin D analog (eg, calcipotriol) and a corticosteroid (eg, betamethasone dipropionate) as the first-line treatment for plaque psoriasis. The latest Cochrane review on treatments for psoriasis [7] reported that the combined use of these drugs from different pharmacological groups is superior in efficacy and safety to their use separately. The combination of 0.005% calcipotriol and 0.064% betamethasone dipropionate has a lower rate of side effects than observed with topical corticosteroids (mainly skin atrophy) and vitamin D analogs (skin irritation and hypercalcemia) used separately.

A new aerosol foam formulation of Cal/BD (Enstilar) for the treatment of plaque psoriasis was introduced in 2017. In an in vitro model of skin penetration of Cal/BD aerosol foam, greater diffusion of both drugs and more stable skin concentrations were demonstrated [8]. The anti-psoriatic effect of Cal/BD aerosol foam was superior to that observed with galenic ointment [9]. Regarding safety, the MUSE study [10] demonstrated a good safety profile, and the PSO-FAST study [11] demonstrated efficacy and tolerability in patients with psoriasis of any severity level.

Adherence to topical treatment depends on multiple factors regarding the product itself and the patient. Cosmetics and speed of action are essential for the patient to correctly comply with the drug dosage. However, other social and even occupational factors can significantly influence adherence. The main objective of this study was to determine the adherence to treatment with Cal/BD aerosol foam in patients with mild–moderate plaque psoriasis 4 weeks after starting treatment. The secondary objectives were to evaluate the: (a) degree of patient satisfaction during and after treatment termination, and (b) treatment’ efficacy, safety, and impact on the patients’ quality of life.

## Methods

### Patients

From November 2017 to November 2019, we recruited patients from the Dermatology Department at San Cecilio University Hospital. Patients were eligible for the study if they were older than 18 years of age and had a clinical diagnosis of plaque psoriasis on the trunk and extremities for at least 6 months, with an affected body surface area (BSA) <10%, a PASI <10, and a Dermatology Life Quality Index (DLQI) score <10. Other inclusion criteria were the ability to apply the treatment or, failing that, to be assisted by personnel who could apply it. Moreover, their scores on the Physician Global Assessment (PGA), modified Investigator’s Global Assessment (mIGA), and modified PASI (excluding the head) had to show at least a slight effect of improvement.

Exclusion criteria were psoriasis in areas other than the trunk and extremities (eg, scalp, face, genitals, and skin folds), a history of allergy to vitamin D analogs or topical corticosteroids, and concurrent or previous treatment with systemic corticosteroids, retinoids (eg, acitretin) or immunosuppressants (eg, methotrexate, cyclosporine, fumaric acid esters) in the past 4 weeks, etanercept (past 4 weeks), adalimumab or infliximab (past 8 weeks), ustekinumab (past 16 weeks), other biologics (past 4 weeks or 5 half-lives), psoralen (past 4 weeks), or UVA or UVB phototherapy (past 2 weeks). Other exclusion criteria were: a likelihood of excessive sun exposure during the study, disorders of calcium metabolism associated with hypercalcemia, skin infections, severe liver or kidney disorders, and a recent diagnosis of other psoriasis types (eg, guttate, erythrodermic, pustular, or exfoliative psoriasis).

### Study Design

This was an open-label, non-placebo controlled, prospective single-center study. The study protocol was approved by the ethics committee of our hospital on 19 oct 2019, with the code Derm_003_2019. The study was carried out in compliance with the principles of the Declaration of Helsinki and good clinical practice. All patients gave written informed consent.

Patients who had previously used antipsoriatic or other relevant treatments underwent a pharmacological “washout period” of at least 4 weeks before starting the study. All patients were educated on how to apply Cal/BD aerosol foam, with the first application directly observed by an investigator. The medication was applied only to the psoriasis plaques of the trunk and extremities, avoiding its use in areas such as the scalp, face, genitals, and skin folds. Cal/BD aerosol foam was applied once a day according to the technical datasheet. Complete resolution of the lesions was considered a reason for discontinuation of the treatment, while the reappearance of lesions allowed the resumption of treatment. Continuous, uninterrupted use of Cal/BD (once per day) was permitted for a maximum period of 4 weeks.

Evaluations were performed at baseline (visit 0) and at each study visit (week 4 and week 12). Adherence to treatment was assessed using the Morisky-Green-Levine scale, 4 weeks and 12 weeks after the start of treatment. The number of cans used by each patient was quantified. The degree of patient satisfaction with the treatment was assessed using the abbreviated Treatment Satisfaction Questionnaire for Medication (TSQM-9) scale at 4 weeks, and at 12 weeks in those patients who restarted treatment due to regrowth of lesions.

The severity of psoriasis was assessed using the mIGA and PGA scales, and the impact on quality of life was assessed using the Psoriasis Disability Index (PDI) and the DLQI. The extent and severity of clinical signs of psoriasis lesions were evaluated using the mPASI. The extent of psoriasis involvement was recorded as a function of the affected body surface area (BSA).

Safety and tolerability were evaluated after 4 weeks of treatment with Cal/BD aerosol foam, and again at 12 weeks in patients who restarted topical treatment due to the regrowth of psoriasis plaques. Serious adverse events and adverse drug reactions were recorded.

### Statistical Analysis

All analyses were performed using the Stata statistical program version 16.0 for MacOS (StataCorp) and the level of significance was set at P < 0.05. Graphics were made using Graph-Pad Prism version 8.0.0 for MacOS (GraphPad Software).

## Results

The study enrolled 65 patients with plaque psoriasis, including 33 men and 32 women, with a mean age of 39.7 years (range, 18–70 years). Regarding the duration of psoriasis, for 36 patients it was less than 5 years, for 9 patients it was between 5 and 10 years, and for 20 patients it was greater than 10 years. No patient had previously undergone treatment with acitretin, 22 patients (33.8%) had used methotrexate, 12 had used cyclosporine, and only 1 patient had undergone biological treatment with etanercept (suspended in 2016 by personal decision). Regarding previous treatments for psoriasis, 40 patients (61.5%) had previously used topical corticosteroids and 39 patients (60%) had previously used Cal/BD gel.

### Treatment Adherence and Satisfaction

**Figure 1:**
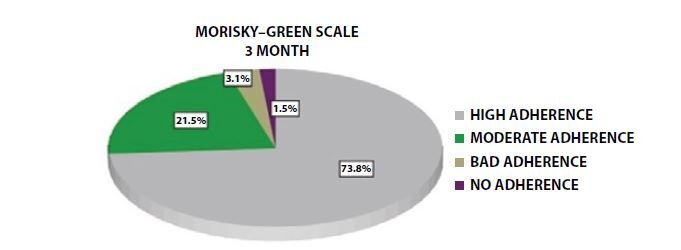
Morisky-Green scores for adherence to Cal/BD treatment at 12 weeks (visit 2). High adherence was observed in 73.8%. Only 4.6% was reported as a non-adherence treatment.

At 4 weeks (visit 1), adherence to treatment with Cal/BD aerosol foam was high on the Morisky-Green-Levine scale in 100% of the participants. At 12 weeks (visit 2), 48 patients (73.8%) showed high adherence, 14 patients (21.5%) had moderate adherence, 2 patients had poor adherence, and 1 patient was not adhering to the treatment (Figure 1).

Patient-reported satisfaction was evaluated using the validated TSQM-9 scale at 4 weeks (visit 1): 46 patients (70.8%) were completely satisfied with the treatment, 8 patients (12.3%) were moderately satisfied, and 11 patients (16.9%) were mildly satisfied. The 12-week score (visit 2) on the TSQM-9 scale showed that 55 patients (84.6%) were completely satisfied with the treatment, 1 patient was moderately satisfied, and 9 patients (13,9%) were mildly satisfied.

**Figure 2:**
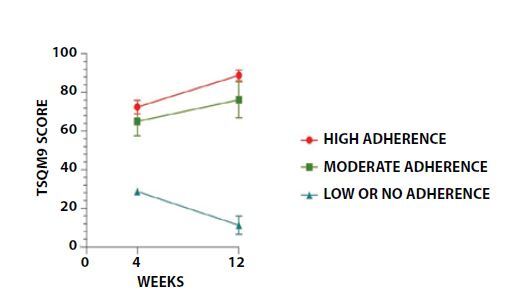
Patient satisfaction with Cal/BD treatment, scored on the TSQM-9 scale, according to treatment adherence. Values are mean and SD

Patients were divided into 3 groups on the basis of their treatment adherence, and the mean TSQM-9 score was calculated for each group (Figure 2). There was a significant difference in TSQM-9 scores between patients who had high adherence to treatment and those who had low or no adherence (t = 32,573; P = 0.007).

A Kruskal-Wallis test was performed to check for significant intergroup differences. A significant relationship between adherence and efficacy was evident, and there were also significant intergroup differences in favor of high adherence in all comparisons. The results are reported in Table 2.

### Efficacy

**Figure 3:**
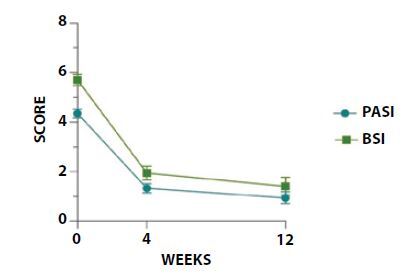
Evolution of psoriasis severity during Cal/BD treatment. Values are mean and SD . PASI = Psoriasis Area and Severity Index; BSA = Body surface area

**Figure 4:**
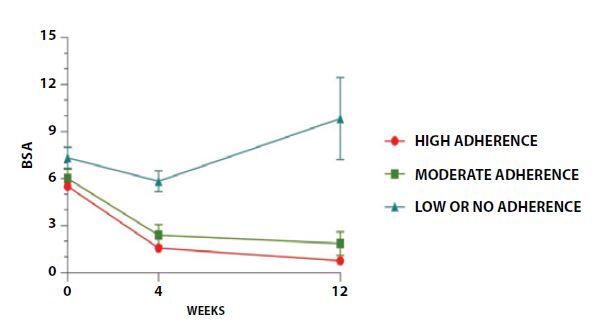
Evolution of psoriasis severity (BSA) according to treatment adherence. Values are mean and SD. BSA = Body surface area

The evolution of psoriasis severity (assessed with PASI, BSA) is shown in Figure 3. The evolution of psoriasis severity (assessed with BSA) according to treatment adherence is shown in Figure 4.

The mean IGA score was 1.954 (SD = 0.891) at the baseline visit, 0.94 (SD = 0.982) at visit 1, and 0.62 (SD = 1.128) at visit 2. The mean PGA score was 2.8 (SD = 0.905) at the baseline visit, 1.19 (SD = 1.211) at visit 1, and 0.77 (SD = 1.1487) at visit 2.

Patients with psoriasis of less than 5 years’ duration had a good response to topical treatment with Cal/BD aerosol foam, with a mean PASI score at week 4 of 0.49 (SD = 0.70) and at week 12 of 0.13 (SD 0.30) (P <0.01). Patients with psoriasis of more than 10 years’ evolution had a poor response to Cal/ BD aerosol foam, with no significant change in PASI scores from week 4 (mean = 2.45; SD = 1.71) to week 12 (mean = 2.15; SD = 2.41; P = 1).

### Quality of Life, Safety and Satisfaction

The mean DLQI score was 10.67 (SD = 4.96) at the baseline visit, 2.41 (SD = 3.87) at visit 1, and 2.24 (SD = 5.25) at visit 2. The mean PDI score was 21.37 (SD = 9.63) at the baseline visit, 5.94 (SD = 7.64) at visit 1, and 4.41 (SD = 9.92) at visit 2.

No patient had side effects related to application of Cal/ BD aerosol foam. Regarding its cosmetic properties, 15.4% responded that the product’s most important attribute was that it did not leave residues or spots on the skin, 7.7% valued the absence of odor, and 76.9% rated it as without data. Patients reported as the most important interest the easy application of the drug to the skin. The overall cosmetic quality of Cal/BD aerosol foam was rated “good” by 90.8% of the participants.

In terms of general satisfaction with Cal/BD aerosol foam, 66.2% of the patients were very satisfied, 15.4% was satisfied, 10.8% was not very satisfied, and 7.7% was not at all satisfied. The patients reporting little or no satisfaction corresponded to those in whom psoriasis did not improve with the treatment.

## Discussion

Stein et al [12], among others, reported that the high efficacy of Cal/BD aerosol foam is an important characteristic in the treatment of mild-moderate plaque psoriasis. Its speed of action, in patients with mild or moderate psoriasis, deserves to be reviewed [13]. This study evaluated the adherence and satisfaction of patients with plaque psoriasis on the trunk and extremities treated with Cal/BD aerosol foam. Over a 4-week period, patients treated with Cal/BD aerosol foam had a significant improvement in disease severity that was directly related to treatment adherence.

Non-compliance with topical therapy continues to be a challenge in clinical practice for patients with psoriasis, and constitutes a key limiting factor in its effectiveness [14]. According to the results obtained using the Morisky-Green-Levine scale, at 12 weeks only 73.8% of the participants were fully compliant with the treatment. This lack of adherence was due to the lack of response to treatment, coinciding in those patients with psoriasis duration of more than 10 years. These patients, who previously had systemic treatment, received treatment with systemic or biological therapy after the study ended.

The reason why therapeutic compliance extends up to 12 weeks is directly related to the effectiveness of Cal/BD aerosol foam beyond the 4 weeks indicated in the datasheet, as demonstrated by Paul et al. [15]. The most frequent difficulties associated with non-compliance are the patients’ perception of the effectiveness of the product and the discomfort of the administration regimen. As observed in our study, both difficulties have been overcome with this new formulation. The quick and effective response to treatment has a direct impact on treatment compliance. Patient expectations are directly linked to therapeutic adherence [16], and treatment failure plays the main role in abandoning therapy, especially in topical treatments.

The secondary objective of the study was to assess patient satisfaction after Cal/BD aerosol foam treatment. The results show how patient satisfaction after treatment was maintained over time. Low satisfaction or dissatisfaction with the treatment, recorded in 9 patients, was justified by their having a partial or no response to the treatment. However, the cosmetic evaluation of the product by these patients was positive.

As previously reported, low adherence to treatment may be justified by the acceptability of the vehicle and its cosmetics [17]. The Cal/BD aerosol foam formula was developed to provide patients with comfortable and easy topical application. This allows for greater adherence to treatment compared with other topical formulations for the treatment of psoriasis. Treatment adherence—an essential objective in chronic skin diseases such as psoriasis—and patient satisfaction are strongly linked, as we have observed in our study.

Another variable recorded during the study was the number of packages used during treatment and follow-up at 12 weeks. Of the participants, 87.6% consumed only 2 cans of Cal/BD aerosol foam. With these data we corroborate what was published by Balak et al. [18] and Duvetorp et al. [19], who considered this medication a cost-effective treatment for plaque psoriasis. Our results regarding treatment adherence, effectiveness, and tolerability of Cal/BD aerosol foam support the conclusions of a recent review of studies on the use of Cal/ BD aerosol foam in clinical practice [20].

The safety of Cal/BD aerosol foam further strengthens its indication as the first line of therapy in patients with mild–moderate plaque psoriasis [21]. In a study, 60% of participants who had previously used Cal/BD gel were asked to compare the two formulations, and 95% said that the new formulation was better, mainly due to its efficacy and cosmetics (non-greasy feel and absence of residue); the remaining 5% considered the gel and foam formulations to be similar in efficacy and cosmetics.

The effectiveness of Cal/BD aerosol foam is essential for adherence to treatment. This drug combination inhibits the production of one of the most important interleukins in psoriasis, IL-17, and decreases its blood level [22]. However, the vasoconstrictive power of the corticosteroid in the new aerosol foam formulation is not superior to that in the gel formulation [23].

This study has limitations due to the small number of enrolled patients. A multicenter study with the participation of specialized psoriasis units could increase the number of patients studied.

## Conclusions

This 12-week study in patients with plaque psoriasis on the trunk and extremities showed that Cal/BD aerosol foam provides a significant improvement in its treatment, with very good adherence and a favorable safety profile. The high level of adherence, the rapid action of the drug, and the adequate cosmetics of the vehicle make Cal/BD in aerosol foam a first-line topical treatment in patients with plaque psoriasis. Cal/ BD aerosol foam can be considered a cost-effective drug for the treatment of plaque psoriasis.

## Figures and Tables

**Table 1:** Characteristics of the 65 Patients with Plaque Psoriasis, at Inclusion

Characteristic	Value
Age, years, mean (SD)	39.7 (12.9)
Sex, n	
Male	33
Female	32
Marital status, n (%)	
Single	21(32.3)
Married	43 (66.2)
Widowed	1 (1.5)
Education, n (%)	
Secondary school	20 (30.8)
High school certi_cate	28 (43.1)
Tertiary	17(26.2)
Occupational status, n (%)	
Working or studying	13 (20.0)
Unemployed or disabled	42 (64.6)
Retired	10 (15.4)
Duration of psoriasis, years mean (SD)	9.5 (9.5)
Previous therapy with topical drugs, n (%)	
Topical corticosteroids	40 (61.5)
Vitamin D_3_ analogs	8 (12.0)
Cal/BD gel (Daivobet)	39 (60.0)
Previous therapy with systemic drugs, n (%)	
Methotrexate	22 (33.8)
Cyclosporine	12 (18.5)
Acitretin	0 (0)
Fumaric acid ester	0 (0)
Biological drugs	1 (1.5)
None	(46.2)

SD = standard deviation.

**Table 2:** Efficacy of treatment according to adherence

	High Adherence (n=48)	Moderate Adherence (n=14)	Low or Null Adherence (n=3)	Total (n=65)
PASI				
T1	4.204 ± 1.409	4.643 ± 1.703	5.333 ± 0.764	4.351 ± 1.465
T 2	1.062 ± 1.278	1.643 ± 1.657	4.167 ± 1.041	1.331 ± 1.498
T3	0.542 ± 1.406	1.107 ± 1.655	6.666 ± 2.887	0.946 ± 1.983
T0-01	−3.142 (−3.541; 2.742)*	−3.000 (−4.080; 1.919)*	−1.167 (−2.601; 0.267)	−3.020 (−3.394; −2.646)*
T0-T2+^	−3.663(−4.147; 3.178)*	−3.536(−4.693; −2.378)*	1.333 (−4.536; 7.203)	−3.405 (−3.909; −2.900)*
BSA				
T1	5.510 ± 1.773	6.000 ± 2.219	7.333 ± 1.155	5.700 ± 1.877
T2	1.573 ± 1.902	2.393 ± 2.543	5.833 ± 1.155	1.946 ± 2.206
T3	0.750 ± 1.910	1.857 ± 2.797	9.833 ± 4.537	1.408 ± 2.934
T0-01+	−3.938 (−4.505; −3.370)*	−3.607 (−4.967; −2.248)*	−1.500 (−1.500; −1.500)*	−3.754 (−4.261; −3.246)*
T0-T2+	−4.760 (−5.359; −4.161)*	−4.143 (−5.590; −2.695)*	2.500 (−8.112: 13.112)*	−4.293 (−4.962; −3.623)*
IGAm				
T1	1.830 ± 0.808	2.070 ± 0.997	3.330 ± 0.577	1.950 ± 0.891
T2	0.750 ± 0.786	1.140 ± 1.167	3.000 ± 0.000	0.940 ± 0.982
T3	0.37 ± 0.815	0.790 ± 1.188	3.670 ± 0.577	0.620 ± 1.128
T0-01	−1.083 (−1.329; −0.838)*	−0.929 (−1.458; −0.399)*	−0.333 (−1.768; 1.101)*	−1.015 (−1.227; −0.803)*
T0-T2+	−1.458 (−1.719; −1.198)*	−1.286 (−1.859; −0.712)*	0.333 (−1.101; 1.768)*	−1.339 (−1.579: −1.097)*
PGA				
T1	2.69 ±0.854	2.930 ± 0.917	4.000 ± 1.000	2.800 ± 0.905
T2	0.98 ± 1.021	1.430 ± 1.453	3.330 ± 0.577	1.180 ± 1.211
T3	0.46 ± 1051	0.930 ± 1.492	5.000 ± 1.000	0.770 ± 1.487
T0-01	−1.708 (−1.969; −1.447)*	−1.500 (−2.169; −0.830)*	−0.667 (−2.101; 0.768)*	−1.615 (−1.854; −1.377)*
T0-T2+^	−2.229(−2.512; −1.947)*	−2.000(−2.679; 1.321)*	1.000 (−1.484; 3.484)*	−2.031 (−2.331; −1.731)*

Data presented as mean ± standard deviation and mean difference (95% confidence interval)

* Friedman test for repeated measure; p <0.001.

+ Kruskal-Wallis test: + intergroup differences between high adherence and low adherence;

^ Intergroup differences moderate adherence vs low adherence; p <0.05.

## References

[1] Parisi R, Symmons DP, Griffiths CE, Ashcroft DM, Identification and Management of Psoriasis and Associated ComorbidiTy (IMPACT) project team (2013). Global epidemiology of psoriasis: a systematic review of incidence and prevalence. J Invest Dermatol.

[2] Boehncke WH, Schön MP (2015). Psoriasis. Lancet.

[3] Michalek IM, Loring B, John SM (2017). A systematic review of worldwide epidemiology of psoriasis. J Eur Acad Dermatol Venereol.

[4] Kimball AB, Gieler U, Linder D, Sampogna F, Warren RB, Augustin M (2010). Psoriasis: is the impairment to a patient’s life cumulative?. J Eur Acad Dermatol Venereol.

[5] Lebwohl M, Ting PT, Koo JY (2005). Psoriasis treatment: traditional therapy. Ann Rheum Dis.

[6] Bewley AP, Shear NH, Calzavara-Pinton PG, Hansen JB, Nyeland ME, Signorovitch J (2019). Calcipotriol plus betamethasone dipropionate aerosol foam vs. apremilast, methotrexate, acitretin or fumaric acid esters for the treatment of plaque psoriasis: a matching-adjusted indirect comparison. J Eur Acad Dermatol Venereol.

[7] Mason AR, Mason J, Cork M, Dooley G, Hancock H (2013). Topical treatments for chronic plaque psoriasis. Cochrane Database Syst Rev.

[8] Basse LH, Olesen M, Lacour JP, Queille-Roussel C (2014). Enhanced in vitro skin penetration and antipsoriatic effect of fixed combination calcipotriol plus betamethasone dipropionate in an innovative foam vehicle. J Invest Dermatol.

[9] Queille-Roussel C, Olesen M, Villumsen J, Lacour JP (2015). Efficacy of an innovative aerosol foam formulation of fixed combination calcipotriol plus betamethasone dipropionate in patients with psoriasis vulgaris. Clin Drug Investig.

[10] Taraska V, Tuppal R, Olesen M, Bang Pedersen C, Papp K (2016). A novel aerosol foam formulation of calcipotriol and betamethasone has no impact on HPA axis and calcium homeostasis in patients with extensive psoriasis vulgaris. J Cutan Med Surg.

[11] Leonardi C, Bagel J, Yamauchi P (2016). The aerosol foam formulation of the fixed combination calcipotriene plus betamethasone dipropionate improves the health-related quality of life in patients with psoriasis vulgaris: results from the randomized PSO-FAST study. J Drugs Dermatol.

[12] Stein Gold L, Lebwohl M, Menter A, Villumsen J, Rosen M, Koo J (2016). Aerosol foam formulation of fixed combination calcipotriene plus betamethasone dipropionate is highly efficacious in patients with psoriasis vulgaris: pooled data from three randomized controlled studies. J Drugs Dermatol.

[13] Pink AE, Jalili A, Berg P (2019). Rapid onset of action of calcipotriol/betamethasone dipropionate cutaneous foam in psoriasis, even in patients with more severe disease. J Eur Acad Dermatol Venereol.

[14] Reich K, Daudén E (2014). Treatment adherence: a hurdle for real-life effectiveness in psoriasis?. J Eur Acad Dermatol Venereol.

[15] Paul C, Stein Gold L, Cambazard F (2017). Calcipotriol plus betamethasone dipropionate aerosol foam provides superior efficacy vs. gel in patients with psoriasis vulgaris: randomized, controlled PSO-ABLE study. J Eur Acad Dermatol Venereol.

[16] Brown KK, Rehmus WE, Kimball AB (2006). Determining the relative importance of patient motivations for nonadherence to topical corticosteroid therapy in psoriasis. J Am Acad Dermatol.

[17] Bewley A, Page B (2011). Maximizing patient adherence for optimal outcomes in psoriasis. J Eur Acad Dermatol Venereol.

[18] Balak DMW, Carrascosa JM, Gregoriou S (2020). Cost per PASI-75 responder of calcipotriol plus betamethasone dipropionate cutaneous foam versus nonbiologic systemic therapies for the treatment of plaque psoriasis in seven European countries. J Dermatolog Treat.

[19] Duvetorp A, Levin LÅ, Engerstedt Mattsson E, Ryttig L (2019). A cost-utility analysis of calcipotriol/betamethasone dipropionate aerosol foam versus ointment for the topical treatment of psoriasis vulgaris in Sweden. Acta Derm Venereol.

[20] Gerdes S, Velasco M, Wu JJ, Hubo M, Veverka KA (2020). Calcipotriol/betamethasone dipropionate aerosol foam for the treatment of psoriasis vulgaris: a review of real-world evidence (RWE). J Dermatolog Treat.

[21] Amat-Samaranch V, Puig L (2020). Safety of calcipotriene and betamethasone dipropionate foam for the treatment of psori asis. Expert Opin Drug Saf.

[22] Røpke M, Bulai Livideanu C, Kaldate R, Snel A, Paul C (2018). Changes in interleukin-17A, macrophage-derived chemokine and adiponectin following treatment of psoriasis with calcipotriol plus betamethasone dipropionate aerosol foam: results from the PSO-ABLE study. Br J Dermatol.

[23] Queille-Roussel C, Nielsen J, Lacour JP (2019). Vasoconstrictor potency of fixed-dose combination calcipotriol (50 μg/g) and betamethasone dipropionate (0.5 mg/g) cutaneous foam versus other topical corticosteroids used to treat psoriasis vulgaris. J Dermatolog Treat.

